# Language considerations for children of parents with substance use disorders

**DOI:** 10.1186/s13011-023-00536-z

**Published:** 2023-05-19

**Authors:** Hannah S. Appleseth, Susette A. Moyers, Erica K. Crockett-Barbera, Micah Hartwell, Stephan Arndt, Julie M. Croff

**Affiliations:** 1 Department of Psychology, Oklahoma State University, Stillwater, OK 74074 USA; 2 Oklahoma State University Center for Health Sciences, Tulsa, OK 74107 USA; 3 Office of Medical Student Research, Oklahoma State University College of Osteopathic Medicine at Cherokee Nation, Tahlequah, OK 74464 USA; 4 Editor Substance Abuse Treatment, Prevention and Policy, Departments of Psychiatry and Biostatistics, College of Medicine, JPP, University of Iowa, Iowa City, IA 52240 USA

**Keywords:** Person-centered language, Children, Stigma, Parent alcohol and drug misuse, Lived experience

## Abstract

Parents with substance use disorders are highly stigmatized by multiple systems (e.g., healthcare, education, legal, social). As a result, they are more likely to experience discrimination and health inequities [1, 2]. Children of parents with substance use disorders often do not fare any better, as they frequently experience stigma and poorer outcomes by association [3, 4]. Calls to action for person-centered language for alcohol and other drug problems have led to improved terminology [5–8]. Despite a long history of stigmatizing, offensive labels such as “children of alcoholics” and “crack babies,” children have been left out of person-centered language initiatives. Children of parents with substance use disorders can feel invisible, shameful, isolated, and forgotten—particularly in treatment settings when programming is centered on the parent [9, 10]. Person-centered language is shown to improve treatment outcomes and reduce stigma [11, 12]. Therefore, we need to adhere to consistent, non-stigmatizing terminology when referencing children of parents with substance use disorders. Most importantly, we must center the voices and preferences of those with lived experience to enact meaningful change and effective resource allocation.

In recent years, there have been many campaigns to use person-centered language to reduce impacts associated with stigmatizing language among individuals with substance use disorders [5, 6, 13]. The goal of these initiatives is to ensure that identity is centered on the individual rather than the disease. Indeed, both the National Institute on Drug Abuse (NIDA) and the National Institute on Alcohol Abuse and Alcoholism (NIAAA) have launched campaigns to center language on individuals and not diagnoses of use disorders (e.g., “words matter” [14], and NIAAA social media posts). NIAAA offered guidelines for applying person-first language when referencing individuals with alcohol use disorder (AUD), which included: (1) describe an individual as “a person with alcohol use disorder” rather than “alcoholic,” and (2) describe an individual as “a person in recovery from alcohol use disorder” instead of “recovering alcoholic.” However, neither NIDA nor NIAAA provided guidelines on person-centered language for children of parents with substance use disorders.

Children of parents with substance use disorders have been excluded from considerations of stigmatizing language —particularly children of parents with AUDs [15]. Children of parents with AUDs have historically been referred to as “children of alcoholics (COA),” “adult children of alcoholics (ACOA),” or “children of substance abusing parents” [16, 17]. All of which perpetuate stigma towards both the parent and child [18]. Indeed, these terms continue to be used even after the inclusion of person-centered language guidelines in the 10th edition of the AMA’s manual of style in 2010. In a recent PubMed search, there were 117 publications using the terms (“children of alcoholics”) OR (“alcoholic parents”) OR (“substance abusing parents”) between 2011 and 2022.

The stigmatization of children of parents with substance use disorders (particularly AUD) has deep roots, some of which can be traced back to the family disease model [19]. Addiction has been described as a “family disease” wherein all family members share an addiction and have their own interacting illness [20]. Briefly, the family disease model proposed that family members are “no longer innocent victims” because they may be acting as “etiological agents or as complicating the illness,” [21]. Many problematic terms arose from this movement such as alcoholic families, codependency, and children of alcoholics [22–24]. Further, US national organizations that were created to provide resources to children of parents with SUD also still hold stigmatizing names such as “the National Association for Children of Addiction” and “Adult Children of Alcoholics and Dysfunctional Families.” The lack of proposed changes to this language has resulted in a mixture of terms being used to describe children with a parent or caregiver with SUD, many of which are stigmatizing and dated.

Children of parents with SUD often experience self-discrimination and disclosure stigma, as well as prejudice from peers, teachers, media sources, and other adults [18, 25]. Dated linguistic choices for describing those with substance use disorders (e.g., addict, junkie, alcoholic, etc.) contribute to increased stigma, barriers to healthcare, and poorer treatment outcomes in affected individuals [26]. Higher levels of social stigma (toward opioid use disorder) has been associated with lower support for public health-oriented policies and increased support for punitive policies [27]. Conversely, the use of person-centered language decreases stigma and improves treatment outcomes for individuals with substance use disorders [26]. Therefore, it is reasonable to assume that the use of person-centered language would likely reduce stigma and improve outcomes for children of parents with SUD. Acknowledging that linguistic practices influence health outcomes and resource allocation, a simple first step is changing the way in which we refer to children of parents with SUD.

Clinical and empirical efforts specifically focused on the needs and protection of children of parents with SUD are long overdue. The lack of uniformity and initiative in changing our language likely contributes to stigma, shame, and exacerbated relational tensions in these individuals. Children of parents with SUD often have strained parental relationships, and these parental relationships may occupy much of the child’s emotional and cognitive bandwidth [10]. They often experience ambivalence toward their parents, feeling both loved and betrayed [28]. Untreated ambivalence toward parents can contribute to children experiencing poorer psychological and physical health as adults [29]. Unfortunately, many do not receive professional support to help them make sense of their emotions, situation, and stressors [10]. If children of parents with SUD do receive professional intervention, it is often focused on their parent’s needs and recovery, rather than the child’s unique experiences and needs. Therefore, language considerations for children of parents with SUD can improve behavioral and health outcomes directly through access to services.

Finally, and most importantly, we must center the preferences of the children and family members of primary caregivers with substance use disorders . As Ziss notes in her commentary about person-centered language, we should not correct the way someone chooses to describe themselves [30]. Further, we must acknowledge that many individuals with substance use disorders and their families do not receive treatment or support resources—which can worsen the associated emotional and economic burden, increase relationship dissatisfaction, and heighten familial instability [31]. These lived experiences may result in family members finding difficulty in developing a neutral or positive view of themselves or their loved ones, which may affect how they label themselves or parents [30]. Nonetheless, it is the responsibility of scientific journals, healthcare providers, research investigators, and media outlets to monitor and enforce professional standards of person-centered language. As a call to action, we suggest that children of parents, or caregivers, with SUD be referred to utilizing the following terms:

- Children of parents with substance use disorders (CPSUD).
- Children of caregivers with substance use disorders (CCSUD).
- Perceived caregiver substance misuse (PCSM).

We strongly recommend that researchers, healthcare providers, clinicians, faculty, and staff in higher education and health institutions adopt standards, implement trainings, and encourage the use person-centered language. Health professionals, healthcare workers, and faculty, for example, should use specific health-based terminology for symptoms and diagnoses of SUD, and learn and teach appropriate communication styles among families with such issues. While many substance-use-focused journals have added requirements for the use person-centered language guidelines within the instructions for authors, there is a need for specific adherence for journals that publish articles focused on pediatric populations—as well as public health, and preventive and family medicine journals. A previous study showed that a journal’s inclusion of its own person-centered guidelines was associated with lower rates of stigmatizing language regarding childhood obesity [32]. While our recommendations focus on the health and scientific communities, through translation this may effectively reach a broader audience, including media dissemination of clinical research and findings. Furthermore, editors, authors, and reviewers of papers need to pay attention to language. As medical researchers and healthcare professionals, it should be our goal to advocate for and amplify the voices and preferences of those with lived experiences—especially regarding children.

## Data Availability

This declaration is not relevant to the content of our submission.

## Declarations

### Ethnical approval

This declaration is not relevant to the content of our submission.
